# Localization of Lipid Raft Proteins to the Plasma Membrane Is a Major Function of the Phospholipid Transfer Protein Sec14

**DOI:** 10.1371/journal.pone.0055388

**Published:** 2013-01-30

**Authors:** Amy J. Curwin, Marissa A. LeBlanc, Gregory D. Fairn, Christopher R. McMaster

**Affiliations:** Department of Pharmacology, Dalhousie University, Halifax, Nova Scotia, Canada; Harvard Medical School, United States of America

## Abstract

The Sec14 protein domain is a conserved tertiary structure that binds hydrophobic ligands. The Sec14 protein from *Saccharomyces cerevisiae* is essential with studies of *S. cerevisiae* Sec14 cellular function facilitated by a sole temperature sensitive allele, *sec14^ts^*. The *sec14^ts^* allele encodes a protein with a point mutation resulting in a single amino acid change, Sec14^G266D^. In this study results from a genome-wide genetic screen, and pharmacological data, provide evidence that the Sec14^G266D^ protein is present at a reduced level compared to wild type Sec14 due to its being targeted to the proteosome. Increased expression of the *sec14^ts^* allele ameliorated growth arrest, but did not restore the defects in membrane accumulation or vesicular transport known to be defective in *sec14^ts^* cells. We determined that trafficking and localization of two well characterized lipid raft resident proteins, Pma1 and Fus-Mid-GFP, were aberrant in *sec14^ts^* cells. Localization of both lipid raft proteins was restored upon increased expression of the *sec14^ts^* allele. We suggest that a major function provided by Sec14 is trafficking and localization of lipid raft proteins.

## Introduction

The Sec14 protein domain, also referred to as the CRAL-TRIO domain, is a conserved tertiary structure that binds hydrophobic ligands in an internal cavity. Mutations in specific Sec14 domain containing proteins in humans result in neurodegeneration, blindness, and cancer, with several disease causing mutations residing in the Sec14 domain itself [1]. The Sec14 protein from *Saccharomyces cerevisiae* is essential, comprised of just the Sec14 domain, and *in vitro* has been demonstrated to extract and transfer phosphatidylcholine (PC) and phosphatidylinositol (PI) between membranes [2], [3], [4]. Studies of *S. cerevisiae* Sec14 function *in vivo* have been facilitated by a sole temperature sensitive allele *sec14^ts^*. This allele contains a mutation resulting in a Gly266Asp conversion, Sec14^G266D^ [2], [3], [5], [6].

The Sec14 protein structure, and that of other Sec14 domain family members, has been solved in liganded and unliganded forms [4], [7], [8], [9]. Opening and closing of a helical gate is required to load and unload phospholipid cargo from the internal phospholipid binding cavity [9], [10]. Ligand loading and unloading by Sec14 is presumed to be relevant to its *in vivo* function. Genetic evidence links alterations in PC and PI metabolism to altered growth of cells with reduced Sec14 function [5], [11], [12], [13], [14], [15], [16], [17], [18], [19], [20]. Inactivation of any of the genes coding for enzymes for PC synthesis restores growth, and the known vesicular trafficking defects, in the absence of the normally essential *SEC14* gene [5], [21]. It is not known how diminution of Sec14 function affects PI metabolism *per se*. What is clear is that when *sec14^ts^* cells are grown at 37°C the level of PI 4-phosphate (PI-4P) decreases by ∼50% [11], [15]. Inactivation of the genes encoding the PI-4P phosphatase *SAC1*, or its activator *KES1* [22], [23], [24], [25], bypass the essential requirement for the *SEC14* gene, and alleviate the known vesicular trafficking defects. In *sec14^ts^* cells grown at 37°C there is an accumulation of intracellular membranes of no obvious organellar source [6], [26]. Investigations into vesicular trafficking defects to date have revealed some export from the *trans*-Golgi, as well as trafficking through endosomes, are defective in *sec14^ts^* containing cells [27].

We performed a genome-wide screen for non-essential *S. cerevisiae* genes whose inactivation would lead to the suppression of the growth defect of *sec14^ts^* cells at 37°C to increase knowledge of cellular processes that intersect with the function of Sec14 in general or the *sec14^ts^* allele specifically (Sec14^G266D^). From this screen we identified 14 genes, three of which were previously known suppressors of the *sec14^ts^* allele, that affect PC and PI-4P metabolism (*PCT1*, *KES1* and *CHO2*) [5], [12], [21]. The remaining 11 genes are new suppressors of *sec14^ts^* function. One of the new genes identified was *RPN4*, which encodes a transcription factor that enhances transcription of genes encoding for subunits of the proteasome [28]. Inactivation of the *RPN4* gene, or pharmacological inhibition of the proteasome, increased the level of the Sec14^G266D^ protein, as did increased expression of the *sec14^ts^* allele encoding the Sec14^G266D^ protein. What was surprising was that under none of these conditions were the known vesicular trafficking defects for the *sec14^ts^* allele restored. Instead, we discovered that trafficking of Fus-Mid-GFP from the Golgi to the plasma membrane, as well as localization of Pma1 an essential plasma membrane proton pump, were defective in *sec14^ts^* cells and restored upon increased expression of the *sec14^ts^* allele. This suggests that proper localization of proteins to lipid rafts is a major function of the phospholipid transfer protein Sec14.

## Results

### A genome-wide genetic screen identifies 14 gene deletions that allow growth of cells containing the *sec14^ts^* allele at the non-permissive temperature of 37°C

Identification of genetic suppressors of the *sec14^ts^* allele has led to the discovery that Sec14 acts at the interface between lipid metabolism and vesicular transport. A search for *sec14^ts^* genetic suppressors has never been done in a genome-wide systematic manner. Using synthetic genetic array (SGA) analysis [29] we sought to identify gene deletions that could allow for growth of cells containing the *sec14^ts^* allele grown at the non-permissive temperature of 37°C. To do so, a haploid strain carrying the *sec14^ts^* allele was crossed with the non-essential yeast gene deletion strain set. The resulting 4795 diploid strains were sporulated and haploids containing the *sec14^ts^* allele and each non-essential gene were isolated and scored for robust growth at 37°C.

The SGA screen was performed three times and genes identified at least two of three times were considered, leading to a total of fourteen gene deletions which could allow growth of *sec14^ts^* cells at 37°C (Table 1). Seven genes have known and direct roles in regulating Golgi function and/or lipid homeostasis, including three genes whose inactivation had been identified previously to restore growth to *sec14^ts^* cells: *PCT1* and *CHO2* that encode enzymes that synthesize the phospholipid PC, and the oxysterol binding protein family member *KES1* [5], [12], [21]. Other genes identified have roles in Golgi function including: *GLO3* encoding an Arf GTPase activating protein (GAP) which regulates retrograde transport between the endoplasmic reticulum (ER) and Golgi [30]; a second regulator of Golgi to ER transport *RER1* [31]; *COG6*, a member of the conserved oligomeric complex that participates in multiple stages of Golgi transport [32]; and, *MNN9* which encodes Golgi resident mannosyltransferase [33].

**Table 1:** *sec14^ts^* suppressing gene deletions.

Gene	ORF	Protein Description	Cellular Process
*PCT1*	YGR202C	Phosphocholine cytidylyltransferase, the rate-determining enzyme of the CDP-choline pathway for phosphatidylcholine synthesis; inhibited by Sec14	Golgi Function/Phospholipid Metabolism
*CHO2*	YGR157W	Phosphatidylethanolamine N-methyltransferase; carries out first methylation step in the phosphatidylcholine biosynthesis pathway	Golgi Function/Phospholipid Metabolism
*KES1*	YPL145C	Member of the oxysterol binding protein family involved in negative regulation of Sec14-dependent Golgi function	Golgi Function/Vesicular Transport
*GLO3*	YER122C	GTPase-activating protein for ADP-ribosylation factors Arf1 and Arf2; involved in retrograde transport between Golgi and ER	Golgi Function/Vesicular Transport
*COG6*	YNL041C	Member of the conserved oligomeric Golgi (COG) complex involved in multiple stages of Golgi transport	Golgi Function/Vesicular Transport
*MNN9*	YPL050C	Protein required for complex N-glycosylation; subunit of the Anp1-Hoc1-Mnn11-Mnn9 mannosyltransferase complex	Golgi Function
*RER1*	YCL001W	Protein involved in retrieval of ER membrane proteins from the early Golgi compartment, including Sec12 (COPII vesicle formation)	Vesicular transport
*CKA2*	YOR061W	Casein kinase II (Protein kinase CK2), catalytic (alpha-prime) subunit	Cell Cycle Progression
*CLB2*	YPR119W	G2/M-specific cyclin, interacts with Cdc28 protein kinase to control events at mitosis; degraded by the anaphase promoting complex	Cell Cycle Progression
*REI1*	YBR267W	Cytoplasmic pre-60S factor; required for the correct recycling of shuttling factors Alb1, Arx1 and Tif6 at the end of the ribosomal large subunit biogenesis; involved in bud growth in the mitotic signaling network	Protein Translation/Cell Cycle Progression
*RPN4*	YDL020C	Transcription factor that stimulates expression of proteasome genes; Rpn4 levels are in turn regulated by the 26S proteasome in a negative feedback control mechanism	Protein Degradation
*NSR1*	YGR159C	Nucleolar protein that binds nuclear localization sequences, required for pre-rRNA processing and ribosome biogenesis	Protein Translation
*XRN1*	YGL173C	Evolutionarily-conserved 5′-3′ exonuclease component of cytoplasmic processing (P) bodies involved in mRNA decay	Protein Translation
*IWR1*	YDL115C	RNA polymerase II (RNAP II) transport factor, conserved from yeast to humans, involved in both basal and regulated transcription from RNAP II promoters, but not itself a transcription factor	Transcription

The remaining seven genes could be grouped in two ways; regulation of cell cycle or protein levels, with one gene falling into both groups. The latter gene, *REI1* was originally identified as a regulator of cell growth (REI =  required for isotropic growth), but more recently was shown to encode a pre-60S cytosolic factor required for ribosomal biogenesis [34], [35]. It was proposed Rei1 could link cell cycle progression and ribosome biogenesis, as bud growth is dependent on high rate of protein synthesis. We also identified two signaling molecules known to regulate cell cycle progression; *CKA2* which encodes a catalytic subunit of protein kinase CK2 (casein kinase II) which has a role in cell survival and cell cycle progression [36], and *CLB2* which encodes a mitotic cyclin that regulates the cyclin dependant kinase, specifically at the end of mitosis [37]. The remaining four genes we classified as regulating protein levels, whether at the level of transcription, translation or turnover. *NSR1* encodes a nucleolar protein involved in ribosome biogenesis [38], *XRN1* encodes a protein involved in mRNA decay [39] and *IWR1* encodes an RNA polymerase II transport factor [40]. The fourth gene of this group identified was *RPN4*, which encodes a transcription factor that increases transcription of genes encoding subunits of the proteosome [41], [42].

### Inactivation of *RPN4* and pharmacological studies provide evidence that the Sec14^G266D^ protein is functional and degraded in a proteasome dependent manner

**Figure 1:**
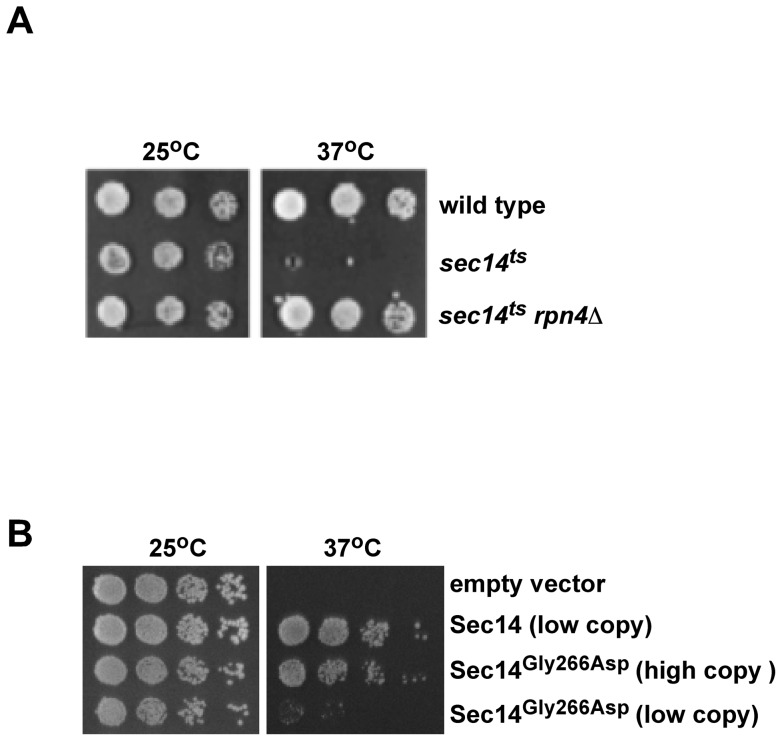
The Sec14 ^G266D^ protein can provide the essential function of Sec14. Serial dilutions of cells cultured at 25°C and then plated and grown at 25°C and 37°C for 3 days. *A*, wild type, *sec14^ts^* and *sec14^ts^ rpn4*Δ. *B*, The *sec14^ts^* strain transformed with either empty vector, a plasmid carried at low copy (ARS/CEN) containing wild type Sec14, or a high copy (2 μ) plasmid containing Sec14^G266D^.

Inactivation of the *RPN4* gene restored growth to *sec14^ts^* cells when grown at the non-permissive temperature for the *sec14^ts^* allele, 37°C (Fig. 1A). The function defective in the *sec14^ts^* encoded protein that prevents growth at 37°C has never been determined. We surmised that inactivation of *RPN4* allowed for growth of *sec14^ts^* cells at 37°C due to decreased proteosome function which in turn resulted in an increase in Sec14^G266D^ protein level. This would also imply that increasing the level of the Sec14^G266D^ protein in and of itself could allow for growth restoration in a *sec14^ts^* yeast strain. Indeed *sec14^ts^* cells with a high copy number (2 μ plasmid, ∼20–30 copies per cell) expressing the *sec14^ts^* allele grew at 37°C, indicating that the Sec14^G266D^ protein can provide the essential function of Sec14 (Fig. 1B).

**Figure 2:**
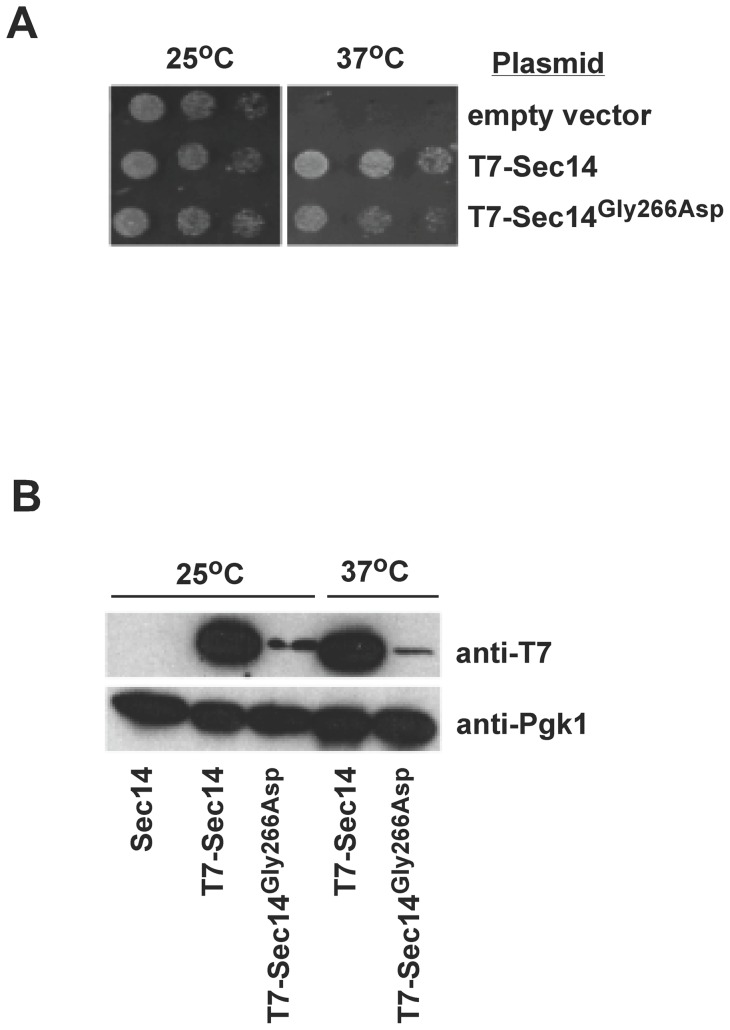
Effect of temperature on Sec14 and Sec14 ^G266D^ protein levels. *A*, the *sec14^ts^* cells were transformed with a plasmid expressing Sec14 containing an N-terminal T7 epitope, untagged Sec14, or empty vector. *SEC14* expression was driven by the constitutive *GPD1* promoter. Cells were grown in solution at 25°C to mid-logarithmic phase, and serial dilutions of identical numbers of cells were spotted onto plates and incubated at 37°C for two days. *B*, cells expressing T7-Sec14 or Sec14^G266D^ were grown to mid-logarithmic phase at 25°C, with a subset shifted to 37°C for 2 hrs. Cells were disrupted by three passes through a French press and membranes were separated from soluble proteins by differential centrifugation. Proteins in each fraction were separated by SDS-PAGE, transferred to PVDF membrane, and western blots were performed. In the blots shown 10 fold more protein extract was loaded in each Sec14^G266D^ lane compared to extracts containing wild type Sec14 for blots versus the T7 epitope due to protein expression level differences.

Next we generated N-terminal T7 tagged versions of wild type Sec14, and Sec14^G266D^ under the control of the high expression promoter of *GPD1*. The wild type and mutant T7-Sec14s were functional as they supported growth of *sec14^ts^* cells at 37°C (Fig. 2A). We examined the protein levels of T7-Sec14 and T7-Sec14^G266D^ in cells grown at 25°C and 37°C. Wild type T7-Sec14 did not change at 25°C versus 37°C while the level of T7-Sec14^G266D^ was significantly lower than T7-Sec14 at both 25°C and 37°C (Fig. 2B). The level of Sec14^G266D^ decreased further when cells were grown at 37°C.

**Figure 3:**
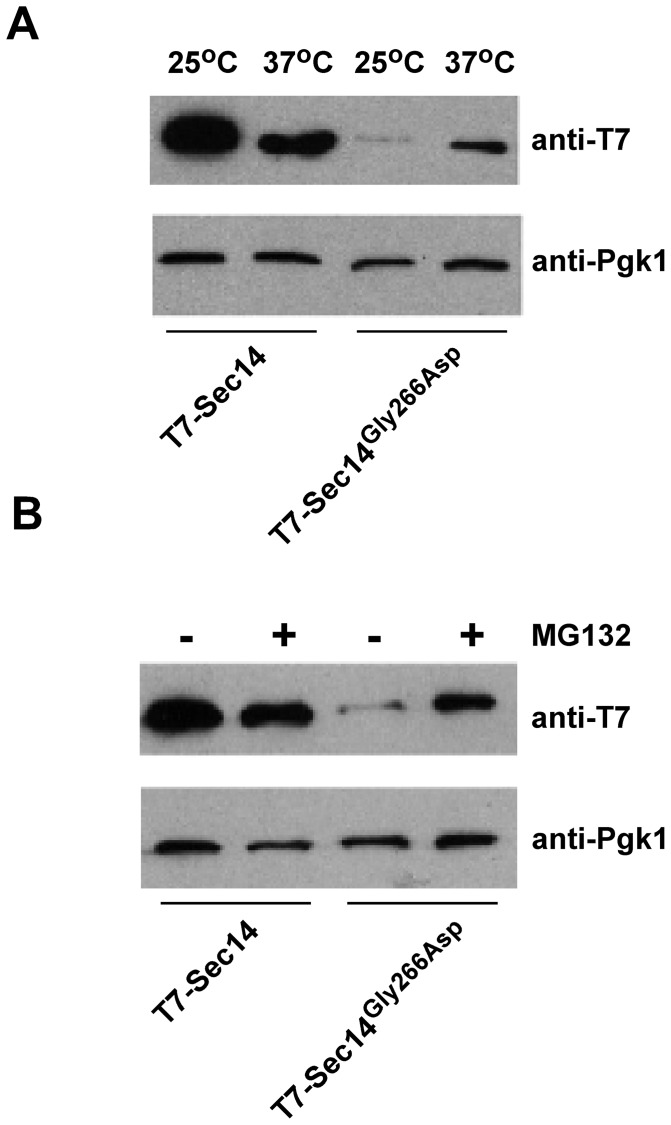
The levels of Sec14^G266D^ is regulated by the proteosome. *A*, the level of Sec14 and Sec14^G266D^ in *sec14^ts^ rpn4*Δ cells. *B*, the level of Sec14 and Sec14^G266D^ in *sec14^ts^* cells treated with MG132. Strains were transformed with plasmids expressing Sec14 or Sec14^G266D^ containing an N-terminal T7 epitope tag and were grown to mid-logarithmic phase at 25°C, with a subset shifted to 37°C for 2 hours (A). For MG132 treatment cells were grown as before and shifted to 37°C in the presence of 100 μM MG132 for 2 hours. Cells were disrupted by three passes through a French press and unbroken cells removed by centrifugation. Protein extract was separated by SDS-PAGE, transferred to PVDF membrane, and western blots versus the T7 epitope were performed. Pgk1 was used as load control.

The T7-Sec14 protein levels were determined in cells lacking *RPN4*. Upon deletion of the *RPN4* gene the level of the T7-Sec14^G266D^ protein did not decrease at 37°C and indeed increased modestly (Fig. 3A), while the level of wild type Sec14 protein did not change. The increased stability of Sec14^G266D^ in the absence of the *RPN4* gene suggests Sec14^G266D^ may be degraded by the proteasome. To test this directly we determined the level of T7-Sec14^G266D^ and T7-Sec14 in the presence of the proteasome inhibitor MG132 in *sec14^ts^* cells also harboring *ise1*Δ, which allows for more efficient uptake of MG132. MG132 treatment increased T7-Sec14^G266D^ level at 37°C, while the level of wild type T7-Sec14 did not change (Fig. 3B). The results are consistent with Sec14^G266D^ being degraded by the proteasome resulting in lower level of Sec14^G266D^.

### Known Sec14 vesicular trafficking pathways are still compromised in cells overexpressing Sec14^G266D^

We assessed the function of the major vesicular trafficking pathways that have been described for *sec14^ts^* cells, expecting that the trafficking defects would be resolved in cells with increased expression of Sec14^G266D^. These included trafficking from the *trans*-Golgi to the plasma membrane by both endosomal and non-endosomal routes, trafficking from the plasma membrane to the vacuole, and analysis of membrane accumulation in cells by electron microscopy. Surprisingly, although increased expression of Sec14^G266D^ restores growth to *sec14^ts^* cells, none of the known vesicular trafficking pathways associated with decreased Sec14 function were alleviated, nor was the accumulation of intracellular membranes.

**Figure 4:**
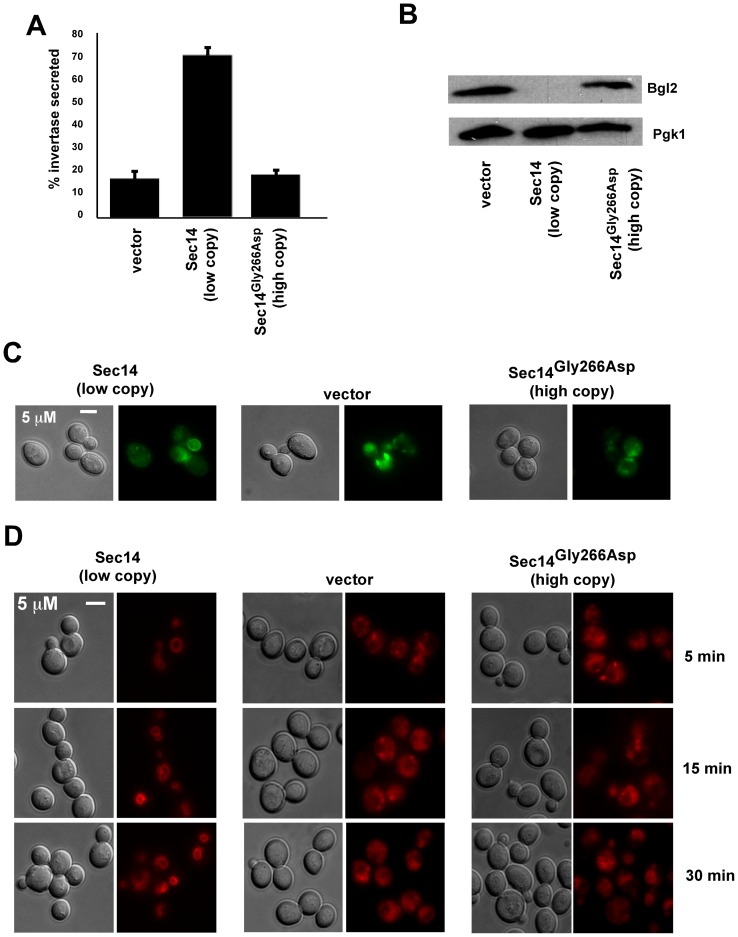
Known vesicular trafficking pathways are still aberrant in growing cells expressing Sec14^G266D^. The *sec14^ts^* strain transformed with either empty vector, a plasmid carried at low copy (ARS/CEN) containing wild type Sec14, or a high copy (2 μ) plasmid containing Sec14^G266D^ were grown at 25°C to mid-logarithmic phase and then transferred to 37°C for 1 hr subsequent to determination of: *A*, invertase secretion (mean ± SE of three separate experiments performed in duplicate), *B*, or internal retention of Bgl2 at 2 and 16 hrs, similar results were seen at both time point with the 2 hr time point shown. *C*, the *sec14^ts^* strain containing plasmid borne GFP-Snc1 was transformed with either empty vector, a plasmid carried at low copy (*ARS/CEN*) containing wild type Sec14, or a high copy (2 μ) plasmid containing Sec14^G266D^. Cells were grown at 25°C to mid-logarithmic phase and then transferred to 37°C for 2 hrs. The localization of GFP-Snc1 was determined by fluorescence microscopy in live cells. *D*, The strains were grown at 25°C to mid-logarithmic phase and then transferred to 37°C for 15 min prior to the addition of FM4-64. The trafficking of FM4-64 in live cells was visualized by fluorescence microscopy.

Secretion from the *trans*-Golgi through both the endosomal route used by invertase and a non-endosomal route used by Bgl2 was not restored. Trafficking through both routes is decreased in *sec14^ts^* cells [5], [13], [20], [27], and increased expression of Sec14^G266D^ did not restore invertase secretion (Fig. 4A), or Bgl2 trafficking to the plasma membrane (Fig. 4B). Expression of wild type Sec14 from a low copy (1–2 copies per cell) plasmid in *sec14^ts^* cells resolved the invertase and Bgl2 secretion defects. Snc1 is a v-SNARE present in vesicles transported from the *trans*-Golgi and the plasma membrane and is recycled back to the *trans*-Golgi to allow for multiple rounds of vesicular trafficking, and growth of *sec14^ts^* cells at the non-permissive temperature of 37°C results in accumulation of GFP-Snc1 in endosomes as well as in the *trans*-Golgi itself [27]. In cells transformed with a 2 μ plasmid expressing Sec14^G266D^ GFP-Snc1 still accumulated in cytoplasmic punctate spots (Fig. 4C). The GFP-Snc1 trafficking defect was relieved by low copy expression of the wild type *SEC14* gene. Trafficking of the lipophillic dye FM4-64 from the plasma membrane to the vacuole is also defective in *sec14^ts^* cells [27] grown at 37°C with FM4-64 accumulating in endosomes in *sec14^ts^* cells (Fig. 4D) [43]. The FM4-64 trafficking defect was not relieved by increased expression of Sec14^G266D^, but was by expression of wild type Sec14.

**Figure 5:**
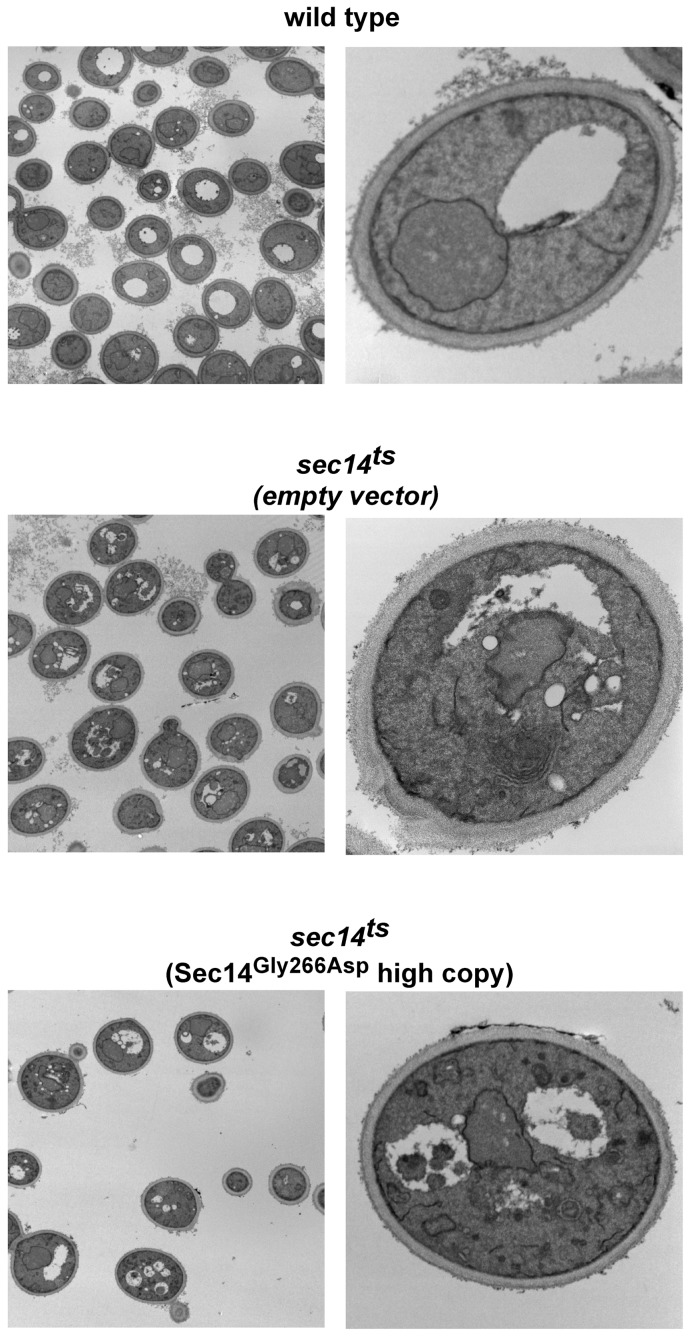
Membranes accumulate in growing cells expressing Sec14^G266D^. Wild type cells and *sec14^ts^* cells containing empty vector, a vector expressing wild type Sec14 on a low copy plasmid, or Sec14^G266D^ on a high copy (2 μ) plasmid, were grown at 25°C to mid-logarithmic phase and an aliquot transferred to 37°C for 1 hr followed by incubation in 1.5% KMnO_4_, 1% sodium periodate, and then 1% NH_4_Cl subsequent to embedding and viewing by transmission electron microscopy.

Consistent with defects in vesicular trafficking still being present, analysis by electron microscopy of *sec14^ts^* cells with increased expression of Sec14^G266D^ revealed an accumulation of membranes at 37°C similar to that observed for cell containing empty vector (Fig. 5). Expression of wild type Sec14 from a low copy plasmid prevented aberrant membrane accumulation in *sec14^ts^* cells. Although increased expression Sec14^G266D^ restored growth to *sec14^ts^* cells, we were unable to observe restoration of the known vesicular trafficking pathways associated with Sec14 function.

### Sec14 is required for trafficking and localization of lipid raft resident proteins

Lipid rafts are domains within membranes enriched in saturated phospholipids, sterols, and sphingolipids. Lipid rafts and lipid raft resident proteins assemble in the Golgi for transport to the plasma membrane. The role of Sec14 in lipid raft resident protein transport has not been determined.

**Figure 6:**
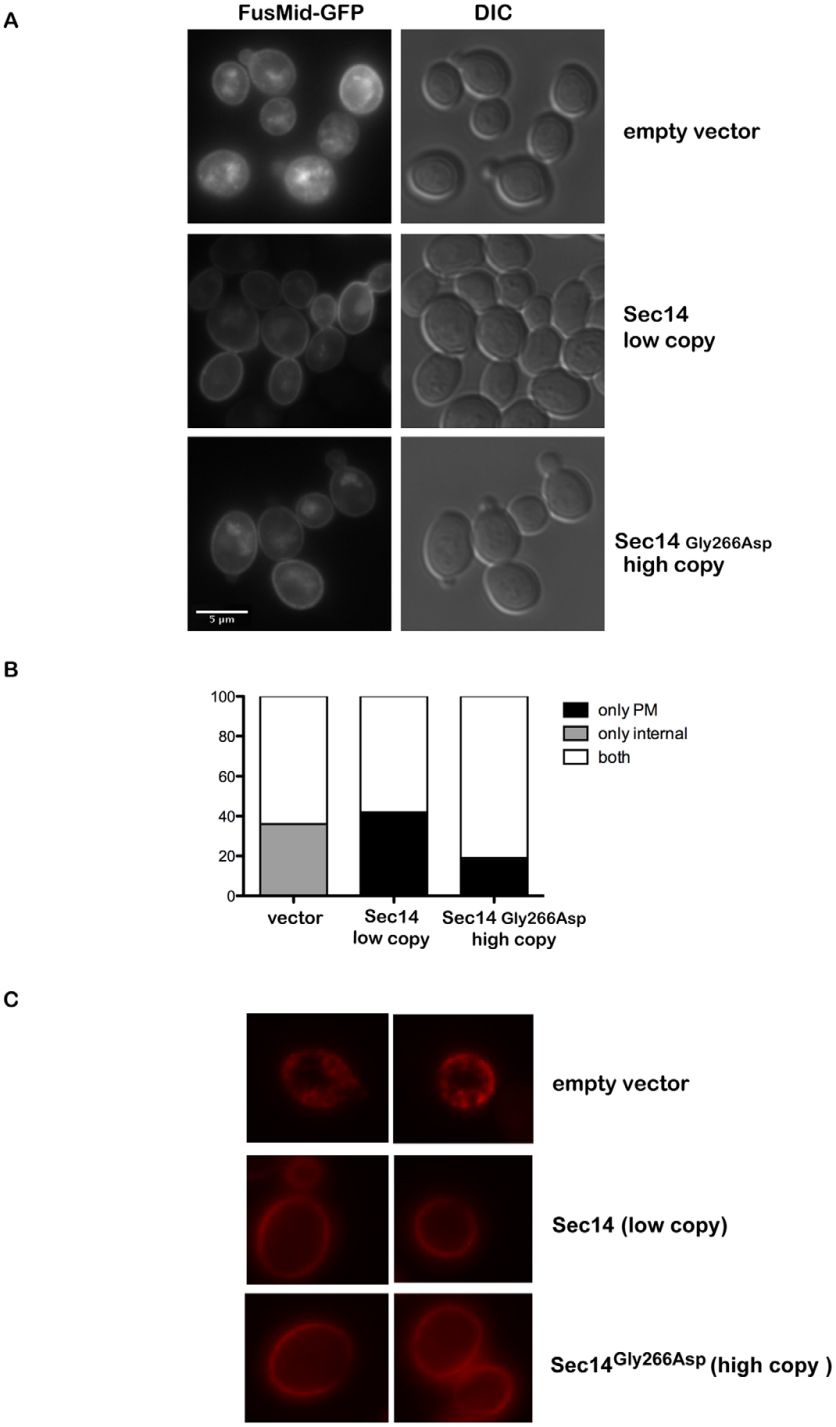
Fus-Mid-GFP and **Pma1 localization is defective in** ***sec14^ts^*** **cells and restored by expression of Sec14^G266D^.** *A, sec14^ts^* cells expressing Fus-Mid-GFP and also containing empty vector, a vector expressing wild type Sec14 on a low copy plasmid, or Sec14^G266D^ on a high copy (2 μ) plasmid, were grown at 25°C in 1% raffinose containing medium to mid-logarithmic phase. Cells were shifted to 37°C in pre-warmed 2% galactose containing medium for 3 hours. *B*, cells from *A* were quantified based on having only plasma membrane (PM) localization, only internal localization or both (vector n = 153, Sec14 n = 73, Sec14^G266D^ n = 107) *C*, the wild type *SEC14* gene was replaced with the *sec14^ts^* allele in a yeast strain expressing chimeric Pma1-RFP. The strain was transformed with either empty vector, a plasmid carried at low copy (ARS/CEN) containing wild type Sec14, and low and high copy (2 μ) plasmids containing Sec14^G266D^. Cells were grown at 25°C to mid-logarithmic phase and then transferred to 37°C for 16 hrs subsequent to determination Pma1-RFP localization by fluorescence microscopy.

To determine if trafficking of lipid raft localized proteins from the Golgi to the plasma membrane was compromised in *sec14^ts^* cells, and if this was restored upon increased expression of the *sec14^ts^* allele, we used the well characterized lipid raft associated protein Fus-Mid-GFP. Fus-Mid-GFP consists of the extracellular region of Fus1p fused to the transmembrane domain and cytoplasmic tail of Mid2p followed by GFP. Fus-Mid-GFP expression is under control of a *GAL* promoter [44] allowing for induction of Fus-Mid-GFP expression upon growth of yeast cells in galactose containing medium to enable trafficking from the Golgi to the plasma membrane of the newly synthesized Fus-Mid-GFP to be determined [29], [44]. Cells were grown at 25°C in medium containing 1% raffinose to logarithmic phase before shifting to pre-warmed (37°C) 2% galacatose containing medium for 3 hours followed by Fus-Mid-GFP localization. In *sec14^ts^* cells expressing empty vector the localization of Fus-Mid-GFP was heterogeneous, with all cells displaying intracellular localization of Fus-Mid-GFP with plasma membrane localization occasionally observed (Fig. 6A). Plasma membrane localization of Fus-Mid-GFP was evident upon expression of high copy *sec14^ts^* Fus-Mid-GFP or expression of the wild type *SEC14* gene from a low copy plasmid, although the localization was quite heterogenous. To quantify Fus-Mid-GFP localization we counted cells based on whether Fus-Mid-GFP was found only in the plasma membrane, only intracellularly, or both, to better determine if there is an effect of increased *sec14^ts^* expression. Cells expressing empty vector showed no plasma membrane only localization, 36% only internal and 64% both (Fig. 6B), while in cells expressing *SEC14* this was somewhat reversed with virtually no cells displaying internal only (1 of 73 cells) and 41% showing only plasma membrane and 58% both. Cells expressing high copy *sec14^ts^* had increased ‘only plasma membrane’ localization at 19% and drastically decreased “internal only” (less than 1%) when compared to cells expressing empty vector where the majority (87%) displayed both intracellular and plasma membrane localization of Fus-Mid-GFP. Therefore Sec14 function seems to contribute to trafficking of this artificial lipid raft cargo. The level of expression from the *GAL* promoter may be too high to completely block the transport from the Golgi, none-the-less, increased expression of the Sec14^G266D^ protein improved the trafficking of Fus-Mid-GFP from the Golgi to the plasma membrane.

To determine if localization of an endogenous lipid raft resident protein was altered in *sec14^ts^* cells, we monitored the localization of the essential proton pump Pma1. Pma1-RFP localization in *sec14^ts^* cells was no longer at the plasma membrane but instead was primarily present in intracellular punctate spots (Fig. 6C). Increased expression of the *sec14^ts^* encoded Sec14^G266D^ protein, or wild type Sec14, restored Pma1-RFP localization. This is the first demonstration of Sec14 participating in the trafficking and localization of lipid raft resident proteins, and indeed it is the trafficking and localization of this class of proteins that is restored upon increased expression of *sec14^ts^* encoded Sec14^G266D^, implying that this may be the essential process defective in *sec14^ts^* cells.

## Discussion

Our genome-wide analysis for suppressors of *sec14^ts^* temperature-sensitivity has led to the identification of a previously unknown role for Sec14, ensuring proper trafficking and localization of lipid raft resident proteins. We suggest this is a major essential function of the phospholipid transfer protein Sec14. Herein, we described that the *sec14^ts^* encoded protein, Sec14^G266D^, was normally present in cells at a lower level than the wild type Sec14 protein, and increasing Sec14^G266D^ level by inhibition of proteasome function or increased dose of the Sec14^G266D^ protein itself, relieved the growth defect of *sec14^ts^* cells. Fus-Mid-GFP and Pma1 localization were defective in *sec14^ts^* cells and restored upon increased expression of Sec14^G266D^, whereas none of the previously identified vesicular trafficking defects associated with loss of Sec14 function were restored, and membrane accumulation still occurred as determined by electron microscopy. Previous work had observed that membrane accumulation was still present in ‘*sec14* bypass suppressor’ cells that had an inactivated *SEC14* gene in combination with inactivating genes for the CDP-choline pathway for PC synthesis, a condition that restored growth to cells lacking Sec14 function [26]. Membrane accumulation and vesicular trafficking defects are clearly present in cells with reduced Sec14 function, but these do not appear to be the major contributing phenotypes to reduced growth as our findings indicate there is no correlation between growth restoration, membrane accumulation, and defects in the vesicular trafficking pathways previously identified as defective *sec14^ts^* cells. Instead, an inability to traffic and localize lipid raft resident proteins appears to be a major function of Sec14.

Three main classes of plasma membrane domains, collectively referred to as lipid rafts, have so far been identified in yeast [29], [34], [35], [39]. The (i) membrane compartment containing Can1 (MCC, also referred to as eisosomes) are enriched in transporters and are thought to have high sterol content, (ii) the membrane compartment containing target of rapamycin kinase complex 2 (MCT) domains have a yet unknown origin, and (iii) the membrane compartment containing Pma1 (MCP) domains. Recently, a fourth highly ordered membrane domain was described that was sterol free and sphingolipid enriched that may play a role in organization of GPI-anchored proteins [40]. Pma1 mislocalization was noted in this study, it will be of interest to assess if Sec14 function also regulates assembly and function of the other types of lipid raft domains.

In this study, we also determined that a reporter of protein assembly into lipid rafts, Fus-Mid-GFP, was compromised in *sec14^ts^* cells and this was partially restored by increased Sec14^G266D^ levels. The Fus-Mid-GFP protein is selectively sorted into sterol and sphingolipid rich domains at the *trans*-Golgi, with this sorting being required for Fus-Mid-GFP trafficking from the Golgi to the plasma membrane, and defects in either sterol or sphingolipid synthesis compromise Fus-Mid-GFP trafficking to the plasma membrane [29], [44]. Prior to Pma1 localization to MCP lipid rafts at the plasma membrane, Pma1 associates with lipid rafts that are forming in the Golgi. Defects in sphingolipid synthesis result in an inability to sort Pma1 into lipid rafts at the Golgi resulting in defective Pma1 trafficking from this organelle [45]. Pma1 can also be mislocalized subsequent to delivery to the plasma membrane due to alterations in its ability to maintain association with MCP lipid rafts once at the plasma membrane [45], [46], [47], [48]. Based on the results from the work presented here, Sec14 may mediate sorting of proteins that are dependent on sphingolipid synthesis for partitioning into lipid rafts at the Golgi for their delivery to the plasma membrane.

Other studies recently linked Sec14 function with sphingolipid metabolism. One study reported that in *sec14^ts^* cells there was a 3–4 fold increase in ceramide mass and changes in the level/metabolism of complex sphingolipids [49]. Another link between Sec14 and sphingolipid metabolism has recently emerged as inactivation of the *SAC1* gene encoding the PI-4P phosphatase can bypass the essential function of Sec14, and the PI produced by Sac1 has been demonstrated to be preferentially used as substrate by Aur1 to convert ceramides into complex sphingolipids [50]. A second gene, *KES1*, can also bypass the essential function of Sec14 and has recently been determined to be an activator of Sac1 PI-4P phosphatase activity [51]. It is clear that sphingolipid metabolism and Sec14 function are linked through both genetic interactions as well as through regulation of sphingolipid levels themselves. Our data imply that regulation of lipid raft protein trafficking and assembly is a node where Sec14 regulation of lipid metabolism and cell function converge.

## Materials and Methods

### Yeast strains and media

The CTY1-1A and CMY503 strains were constructed as described [5], [27]. Other strains used in this study were constructed using standard yeast molecular genetic techniques (Table 2). Rich medium was yeast extract protein dextrose (YEPD, 1% bacto-yeast extract, 2% bacto-peptone, 2% dextrose). Minimal medium was synthetic complete (SC, 0.67% bacto-yeast nitrogen base without amino acids, 2% dextrose, and nutrients as required for nutrient auxotrophies and plasmid selection).

**Table 2:** Yeast strains used in this study.

Strain	Genotype	Source
CTY1-1A	**a** *ura3 his3 lys2 sec14^ts^*	(5)
CMY503	α *mfa1*Δ::*MFA1pr-HIS3 can1*Δ*0 his3*Δ*1 leu2*Δ*0 ura3*Δ*0 lys2*Δ*0 sec14^ts^-NatMx4*	(21)
BY4741	**a** *his3*Δ*1 leu2*Δ*0 ura3*Δ*0 0 met15*Δ*0*	Euroscarf
CMY505	**a** *his3*Δ*1 leu2*Δ*0 ura3*Δ*0 0 met15*Δ*0 sec14^ts^-NatMx4*	(21)
*SNF7*-RFP	α *his3*Δ*1 leu2*Δ*0 lys2*Δ*0 ura3*Δ*0 SNF7-*RFP-*KanMx4*	E. O'Shea
CMY566	α *his3*Δ*1 leu2*Δ*0 lys2*Δ*0 ura3*Δ*0 SNF7-*RFP-*KanMx4 sec14^ts^-NatMx4*	This study
*CHC1-*RFP	α *his3*Δ*1 leu2*Δ*0 lys2*Δ*0 ura3*Δ*0 CHC1-*RFP-*KanMx4*	E. O'Shea
CMY557	α *his3 leu2 lys2 ura3 CHC1-*RFP-*KanMx4 sec14^ts^-NatMx4*	This study
*ANP1-*RFP	α *his3*Δ*1 leu2*Δ*0 lys2*Δ*0 ura3*Δ*0 ANP1-*RFP-*KanMx4*	E. O'Shea
CMY559b	α *his3 leu2 lys2 ura3 ANP1-*RFP-*KanMx4 sec14^ts^-NatMx4*	This study
*ise1*Δ	**a** *his3*Δ*1 leu2*Δ*0 ura3*Δ*0 lys2*Δ*0 ise1*Δ*::KanMx4*	Euroscarf
CMY570	**a** *his3*Δ*1 leu2*Δ*0 ura3*Δ*0 lys2*Δ*0 ise1*Δ*::KanMx4 sec14^ts^-NatMx4*	This study
*rpn4*Δ	**a** *his3*Δ*1 leu2*Δ*0 ura3*Δ*0 lys2*Δ*0 rpn4*Δ*::KanMx4*	Euroscarf
CMY571	**a** *his3*Δ*1 leu2*Δ*0 ura3*Δ*0 lys2*Δ*0 rpn4*Δ*::KanMx4 sec14^ts^-NatMx4*	This study

For cell growth assays, cells were grown to mid logarithmic phase, cell concentration was determined by measuring OD_600 nm_, cells were concentrated to 0.1 absorbance units per ml, a series of serial dilutions were plated on solid medium, and cells were grown for 2–3 days at the indicated temperatures.

### Plasmid construction

The *sec14^ts^* plasmids were constructed by site-directed mutagenesis of a plasmid-borne wild type *SEC14* gene to convert Gly^266^ to Asp, followed by subcloning into low copy pRS415 (CEN/ARS) or high copy pRS425 (2 μ) plasmids [52].

*SEC14* and *sec14^ts^* versions with an N-terminal T7 epitope under the control of the glycerol phosphate dehydrogenase (*GPD1*) promoter were constructed using PCR to amplify the open reading frames of plasmid borne *SEC14* or *sec14^ts^* using primers 5′-GACTGAGAATTCATGGCTAGCATGACTGGTGGA-3′ and 5-GACTGAGTCGACTCATTTCATCGAAAAGGCTTCCGG-3′, TA cloning into pCR-Topo2.1 (Invitrogen), restriction enzyme digestion using EcoRI and SalI, ligation into pET23a (Novagen) to add the N-terminal T7 epitope, followed by subcloning into the yeast shuttle vector p416-GPD [53].

### Synthetic genetic array (SGA) screen

The SGA genetic screen was performed essentially as described [54], [55] with the below modifications. CMY503 (containing the temperature sensitive *SEC14* allele, *sec14^ts^*) was mated with 4,795 *S. cerevisiae* single gene deletion strains at 25°C, diploids were selected, and cells were sporulated for 5 days at 25°C. To ensure that the haploid cells obtained were from mated diploids, cells were selected for histidine prototrophy followed by growth on medium containing G418 and nourseothricin (Nat). The resulting haploids were incubated at 25°C or 37°C. Three independent screens were performed and mutants whose inactivation resulted in growth at 37°C when in combination with the *sec14^ts^* allele in at least two of three screens were considered for further analysis. Genetic interactions were confirmed by a combination of random spore analysis, tetrad dissection, and isogenic strain construction.

### Vesicular trafficking assays

Cells containing GFP-Snc1 or Pma1-RFP were grown to early log phase at 25°C in SC medium, an aliquot shifted to 37°C, and live cells imaged using DIC and fluorescence microscopy (using the GFP filter) of a Zeiss Axiovert 200 M microscope fitted with a plan-neofluor 100x oil immersion lens at the indicated time points. Internalization of the lipophillic dye FM4-64 [43] was performed on cells grown to mid-logarithmic phase in SC medium at 25°C or cells that were resuspended in pre-warmed 37°C medium and grown at 37°C for 15 minutes. Cells were subsequently labeled with 40 μM FM4-64 in DMSO (or DMSO control) for 2 minutes, washed with medium, and incubated further with live cells were imaged at the indicated time points using a rhodamine filter. For imaging Fus-Mid-GFP, cells were grown to logarithmic phase in 2% raffinose containing medium and shifted to pre-warmed 37°C 2% galactose containing medium and further incubated at 37°C for 3 hours. Live cells were imaged with Leica DMI6000B microscope equipped with a DFC 360 FX camera using a HCX Pl APO 100X 1.4 objective. Images were taken using Leica LAS AF software.

Bgl2 secretion was determined essentially as described [56]. Mid-log phase cells were incubated at 25°C with half the culture shifted to 37°C for 1–16 hrs. Cells were harvested by centrifugation, resuspended in 10 mM NaN_3_, 10 mM KF, and incubated on ice for 10 min. Cells were centrifuged at 10,000× *g* for 1 min and pellets resuspended in ice cold 100 mM Tris–H_2_SO_4_, pH 9.4; 50 mM ß-mercaptoethanol; 10 mM NaN_3_; 10 mM KF, incubated on ice for 15 min, centrifuged as before, washed with 0.5 mL spheroplast buffer (50 mM KH_2_PO_4_–KOH, pH 7; 1.4 M sorbitol; 10 mM NaN_3_), and pelleted. Cells were resuspended in spheroplast buffer containing 167 μg/mL zymolyase 100T and incubated for 30 min at 25°C. Spheroplasts were then pelleted at 5,000× *g* for 10 min and resuspended in 2x SDS–PAGE sample buffer. Proteins were separated using 10% SDS–PAGE and Bgl2 was detected by western blot using a rabbit polyclonal antibody against Bgl2. Bgl2 antibodies were the kind gifts of Randy Schekman (University of California, Berkeley) and Wei Guo (University of Pennsylvania). Invertase secretion index was determined as described [57].

### Electron microscopy

Cells were grown to mid logarithmic phase, half the culture was shifted to 37°C for various time points, incubated in 1.5% KMnO_4_, followed by 1% sodium periodate and then 1% NH_4_Cl. Embedding, image capture and processing was performed by the Electron Microscopy Facility in the Faculty of Medicine at Dalhousie University using standard procedures.
